# Dentistry in the District of Columbia

**Published:** 1880-02

**Authors:** 


*Dentistry in the District of Columbia.*—Senator Vance, of the Senate District committee, United States Congress, has introduced a bill to regulate dentistry in the District and "for the protection of the people of the District." The bill makes it unlawful for any person to practice dentistry in the District, without a diploma from some dental college, duly and legally incorporated, or a certificate from the Washington Dental Society; but the provisions shall not apply to persons now practicing in the District until April 1st, 1881; but no person shall, after the passage of the act, begin the practice here, except under the act. Any person violating the law is made guilty of a misdemeanor and subject to a fine of not less than $50 nor more than $200, and in default imprisonment from 30 to 90 days. The act does not prohibit physicians and surgeons from extracting teeth. The Washington City Dental Society shall immediately, on the passage of the act, and at its annual meeting in December each year, appoint five members to constitute a board of examiners, and they shall examine all persons desiring to practice dentistry and to those qualified they shall give a certificate to that effect; but a regular diploma shall be *prima facie* evidence of competency. A majority of the board shall be required to make an examination and sign a certificate. The names of the board must be published for two weeks after each appointment of a new board. A fee of $5 shall be charged each applicant for examination, the proceeds to pay the expenses of the act, but the members of the board receive no compensation for their services. The act goes into effect upon its passage.

A Bill, published elsewhere, has been framed for passage by the Maryland Legislature. In both, the latter State and the District of Columbia, are a number of irregular practitioners of dentistry whose course will come under the provisions of the proposed law. H.

